# Hybrid Polylactic-Acid–Pectin Aerogels: Synthesis, Structural Properties, and Drug Release

**DOI:** 10.3390/polym15020407

**Published:** 2023-01-12

**Authors:** Gabrijela Horvat, Klara Žvab, Željko Knez, Zoran Novak

**Affiliations:** Faculty of Chemistry and Chemical Engineering, University of Maribor, SI-2000 Maribor, Slovenia

**Keywords:** hybrid aerogels, bioaerogel, pectin, polylactic acid, wound healing

## Abstract

Wound-dressing materials often include other materials stimulating wound healing. This research describes the first formulation of biodegradable hybrid aerogels composed of polylactic acid and pectin. The prepared hybrid material showed a highly porous structure with a surface area of 166 ± 22.6 m^2^·g^−1^. The addition of polylactic acid may have decreased the surface area of the pure pectin aerogel, but it improved the stability of the material in simulated body fluid (SBF). The pure pectin aerogel showed a high swelling and degradation ratio after 3 h. The addition of the polylactic acid prolonged its stability in the simulated body fluid from 24 h to more than one week, depending on the amount of polylactic acid. Biodegradable aerogels were loaded with indomethacin and diclofenac sodium as model drugs. The entrapment efficiencies were 63.4% and 62.6% for indomethacin and diclofenac sodium, respectively. Dissolution of both drugs was prolonged up to 2 days. Finally, sodium percarbonate and calcium peroxide were incorporated into the bioaerogels as chemical oxygen sources, to evaluate oxygen generation for potential wound healing applications.

## 1. Introduction

Wounds are skin defects caused by physical or thermal damage, and are classified based on the time of recovery [1]. Acute wounds typically can recover in days, but chronic wounds can take much longer, and treating such wounds is often challenging. The healing process often results from a correct approach to wound treatment. Management of wounds accounts for assessing the wounds and the patient, debridement, and dressing [1]. Wound dressings are, nowadays, often specially designed for a particular wound type. Standard dressings such as gauze and bandages offer only physical protection, and do not have any advancements in the case of wound healing. Modern dressings have better biocompatibility, degradability, and moisture retention [2]. They need to keep the wound from dehydration and promote the recovery of the wound [3]. Among the most-researched novel wound dressings are hydrogels, three-dimensional structures with extraordinary properties such as swelling and water absorption. However, hydrogels are not stable at room conditions; therefore, they should be dried either at room temperature and pressure, by freeze drying, or by supercritical drying. The latter offers a significant advantage over other drying methods, since there are no capillary forces in the supercritical region. Therefore, the final material, aerogel, has the same structure as the initial hydrogel. Aerogels are porous solids with large surface areas and high water uptake, and are often used as drug carriers or absorbents. Biobased aerogels are commonly prepared from polysaccharides or proteins and other biopolymers.

Even though the research on aerogels for wound-healing applications is still in the initial phase, essential developments have been accomplished in recent years highlighting the value of these materials in wound healing and regeneration [4]. 

Polymer-based dressings have already been employed for controlled drug delivery to wounds [5,6,7,8,9]. Pectin is a natural polysaccharide that is well known for its gelation properties. After forming hydrogels, it can easily be transformed into a more stable, dry form of aerogel [10,11,12]. Pectin has a high swelling ability, and its fluid absorption and acidity enhance the bacterial or viral barrier properties of wound dressings [13]. Its combination with polylactic acid (PLA) may be beneficial in terms of stability and better mechanical properties. PLA is biodegradable, biocompatible, and has low immunogenicity. It is an excellent platform for incorporating various compounds such as drugs, antibiotics, peptides, etc. [14]. One of the significant drawbacks of PLA is its hydrophobicity, which limits its water uptake capacity [15]. Therefore, to prepare a wound-dressing material with better stability and higher water uptake capacity, the combination of pectin with PLA shows a promising outcome. 

For faster wound treatment, the wound dressing is not enough, and often novel wound dressings contain other components, such as drugs, which may benefit wound healing. Using non-steroidal anti-inflammatory (NSAID) medications in wound dressings can reduce pain, which causes the release of hormones such as cortisol and norepinephrine, and, consequently, speeds up the healing process [16]. Therefore, reducing pain may shorten the healing time significantly [17]. Even though NSAIDs are mostly taken orally, several researchers have reported their integration into different wound-dressing formulations [18,19,20]. Diclofenac sodium (DCF) is an NSAID that can relieve pain and swelling by targeting the inflammatory phase of wound healing [21]. Indomethacin (IND) is another NSAID that blocks cyclooxygenase in the body, which is responsible for producing irritant chemicals in response to injury. Thus, by blocking this substance, indomethacin reduces symptoms of pain and inflammation [22]. As opposed to NSAIDs, which may benefit the healing process by reducing pain and inflammation, oxygen-generating materials provide oxygen to the cells of the ischemic tissue so they can survive, proliferate, and function. Therefore, oxygen-generating materials support tissue healing and regeneration [23]. Aerogels, with their porous structures, can incorporate different drugs or other compounds and release them in the desired manner [24,25].

This study aimed to design an aerogel-based wound-dressing material with incorporated NSAID and oxygen-generating compounds, to study its properties and performance in drug release. Pectin–PLA aerogels were prepared for the first time, incorporated with two NSAID drugs, DCF and IND, and with oxygen-generating agents, sodium percarbonate (SPO) and calcium peroxide (CPO).

## 2. Materials and Methods

The following chemical reagents were obtained: pectin (from citrus, TCI Europe), polylactic acid (PLA, Goodfellow, granular, 3 mm nominal granule size, Sigma Aldrich), ethyl lactate ((S)-(-)-ethyl lactate for synthesis, Merck KGaA, Sigma Aldrich), ethanol (ACS reagent, ≥99.5%, Sigma Aldrich), indomethacin (analytical grade, ≥99% purity, Sigma Aldrich), diclofenac sodium salt (diclofenac, 10 µg/mL in acetonitrile, 98.0% purity, CHEMOS GmbH & Co. KG), calcium peroxide (75%, −200 mesh, Sigma Aldrich), sodium percarbonate (avail. H_2_O_2_ 20–30%, Sigma Aldrich). All other chemical reagents were of analytical grade (Sigma Aldrich), and deionized water was used throughout.

### 2.1. PLA–Pectin Aerogel Preparation

To prepare the aqueous pectin solution, pectin was added slowly to water while stirring to avoid clumps, and mixed for an additional 2 h. The PLA was dissolved in ethyl lactate (EL) at 120 °C until homogenization. The PLA solution was poured into the aqueous pectin solution while stirring, and then their homogeneous solution was left to form a gel. The wet gel was then washed with ethanol and stored in ethanol until the supercritical drying of samples. Three different ratios of pectin:PLA were used for final aerogel preparation and are summarized in Table 1.

### 2.2. Drug-Loaded Aerogel Preparation

Diclofenac sodium and indomethacin were incorporated into the P1:PLA1 sample.

Diclofenac sodium is a water-soluble drug and belongs to BSC Class II [26]. Therefore, 500 mg of diclofenac sodium was added to the initial pectin solution, and then the PLA solution in ethyl lactate was added as described in 2.1. Before supercritical drying, the sample was stored in ethanol saturated with diclofenac sodium. 

Indomethacin is a low-water-soluble drug and belongs to BSC Class II [27]. A total of 500 mg of indomethacin was added to the PLA/EL solution. This mixture was then added to an aqueous pectin solution, and, after the gel was formed, the sample was stored in ethanol saturated with indomethacin, before supercritical drying.

### 2.3. Oxygen-Generating Aerogel Preparation

Sodium percarbonate (SPO) and calcium peroxide (CPO) were chosen as oxygen generation sources. First, an aqueous pectin solution was prepared, as described in 2.1. The PLA was dissolved in EL, and then SPO (1.0 g) and CPO (1.0 g) were added to this solution. After the complete dissolution of PLA, SPO, and CPO in EL, this solution was transferred to an aqueous pectin solution. After the gel was formed, it was transferred immediately to ethanol to stop the initial production of oxygen after water contact. Finally, the samples were dried supercritically.

### 2.4. Reference Aerogel Preparation

Pectin and PLA aerogels were prepared for comparison with hybrid materials. The pectin aqueous solution was prepared by stirring pectin in water for at least 2 h. Then, ethyl lactate was added according to Table 1 and the gel was stored in ethanol before supercritical drying. 

The PLA aerogel was prepared by dissolving the PLA completely in ethyl lactate at 120 °C. Then, water was added according to Table 1, and the gel was stored in ethanol before supercritical drying. 

### 2.5. Supercritical Drying

Supercritical drying was performed on a supercritical extraction unit (Figure S1) by placing the sample in the 500 mL autoclave, filled with ethanol, and then heating it to 42 °C. After reaching the set temperature the pressure was raised to 120 bar (a pressurization rate of 1 bar min^−1^). Ethanol extraction was performed for 6 h at a CO_2_ rate of approximately 200 L h^−1^ at normal conditions. Finally, the system was slowly depressurized and let cool down. 

### 2.6. Characterization

Nitrogen adsorption measurements ASAP 2020 (Micromeritics Instrument, Norcross, GA, USA) were performed to determine the surface area (BET), pore size distribution, and average pore size (BJH desorption method). The aerogels were degassed before the analysis for 10 h at 70 °C, and then analyzed with nitrogen at −196 °C. The specific surface area of the samples was calculated by applying the BET theory to the nitrogen adsorption data within the 0.06–0.30 P/P0 range. To understand the materials’ structure better, we obtained micrographs with a field emission scanning electron microscope (FE-SEM) Sirion 400 NC (FEI, Eindhoven, The Netherlands). The samples were sputter-coated with gold particles and fixed to aluminum sample holders with double-sided carbon tape. Then they were scanned at an accelerating voltage of 10 kV using a WD detector. The density of the materials was determined by measuring the volume of the sample with a digital caliper (Mitutoyo (UK) Ltd., Andover, UK) and weighing with a high precision balance (XPR206DR/M, Mettler Toledo, Zürich, Switzerland)

The thermal transitions were studied using a differential scanning calorimeter (DSC) within an N_2_ atmosphere with a 10 °C min^−1^ heating rate. The analysis was performed on an HP DSC1 Mettler Toledo apparatus (TGA/DSC1, Mettler Toledo AG (MTANA), Zürich, Switzerland). The temperature range of the analysis was set at 20–600 °C. ATR-FTIR spectra of the PLA, EC-PLA, and EC were recorded using an IRAffinity-a FTIR spectrometer (Shimadzu, Japan) with an attenuated total reflection (ATR) module at a scan range of 4000–600 cm^−1^. Near-infrared (NIR) spectra were recorded on a Fourier-transform near-infrared spectrometer (Matrix-F, Bruker Optics, Ettlingen, Germany) from 12,000 to 4000 cm^−1^ in absorbance mode. Sixty-four scans were obtained for each sample, with a spectral resolution of 4 cm^−1^ and then averaged. The presented spectra are the average of 32 consecutive measurements. 

### 2.7. Swelling and Stability

Structural stability and integrity were evaluated for 102 h at 37 °C. The samples were immersed in simulated body fluid (SBF) with ion concentrations of 142.0 mM Na^+^, 5.0 mM K^+^, 1.5 mM Mg^2+^, 2.5 mM Ca^2+^, 148.8 mM Cl^-^, 4.2 mM HCO_3_^-^, 1.0 mM HPO_4_^2-^, and 0.5 mM SO_4_^2-^. The pH of the SBF was adjusted to pH 7.40 at 36.5°C with 1M HCl. The wet weight was measured after 15 min equilibration, and at 1, 3, 8, 25, 48, 72, and 102 h of immersion. The weight variation was calculated using Equation (1):

(1) $$weight\;variation\;\left(\%\right)=\left(1-\frac{\;w_{tn}}{w_{15min}}\right)\times 100\%$$

where $w_{tn}$ and $w_{15min}$ are the wet weights of the samples at the defined time and after 15 min of swelling, respectively.

### 2.8. Drug Content and Entrapment Efficiency

The drug-loaded samples were immersed in 250 mL ethanol in a sealed beaker during constant stirring at 500 rpm. After 72 h the amounts of diclofenac sodium and indomethacin were evaluated using UV spectrometry (Varian, Cary 50 Probe UV spectrophotometer, Agilent Technologies) at 284 nm and 245 nm, respectively. The calibration curve determined the mass of the drug ($m_{d}$). The drug content (DC) was calculated from Equation (2):

(2) $$DC\;\left[\%\right]=\frac{m_{d}}{m_{s}}\times 100\%$$

where $m_{d}$ is the mass of the drug (mg), determined spectrophotometrically from the calibration curve, and $m_{s}$ is the initial mass of the weighted aerogel sample. Each test was made in triplicate, and the average values were calculated with standard deviations.

Entrapment efficiency represents the ratio between the amount of the incorporated drug detected in the aerogel and the initial amount of the added drug. The entrapment efficiency was calculated with Equation (3):

(3) $$EE\;\left[\%\right]=\frac{m_{d}}{m_{i}}\times 100\%$$

where $m_{d}$ is the mass of the drug (mg), determined spectrophotometrically from the calibration curve, and $m_{i}$ is the initial amount of the drug (mg) that was added to a sol-gel solution before the gelation step. The test was performed in triplicate.

### 2.9. In Vitro Drug Release

For the in vitro drug release testing, simulated body fluid (SBF) was prepared with an ion concentration as listed above (Section 2.7). The drug dissolution test was performed in a sealed beaker at 37 ± 0.5 °C while stirring at 50 rpm. P1:PLA1:IND and P1:PLA1:DCF samples were placed in 50 mL SBF with pH 7.4. Aliquots (1 mL) were withdrawn and filtered before being analyzed spectrophotometrically at 275 nm for DCF sodium and 265 nm for IND. Sampling was performed after 0.25, 0.5, 0.45, 1, 1.5, 2, 4, 7.5, 24, and 48 h. The cumulative release was calculated with Equation (4).

(4) $$cumulative\;release\;\left[\%\right]=\frac{c\cdot V}{m_{t}}\times 100\%$$

where *c* is the concentration of the IND or DCF in the release medium after the selected time intervals in mg/mL, *V* is the volume of the release medium in mL, and $m_{t}$ is the total amount of the drug within the release medium in mg, obtained 48 h after the dissolving test. The drug dissolution test was performed in triplicate.

### 2.10. Oxygen Release from Hybrid Aerogels

Oxygen generation from P1:PLA1:Ox aerogels was determined using the standard water displacement method. The aerogel was placed in a 50 mL syringe (Chirana T. Injecta, A.S., Stará Turá, Slovakia) filled with 10 mL of ultrapure water. Then, the 50 mL syringe was connected to a 10 mL syringe (Becton Dickinson, S.A., San Agustín del Guadalix, Spain) containing 5 mL ultrapure water through a 23 G-1 needle (Chirana T.Injecta, A.S., Stará Turá, Slovakia).

The amount of water displaced in the 10 mL syringe was measured at predetermined time points for 2 days to measure the oxygen release from the aerogel. The amount of water displaced is directly proportional to the amount of oxygen generated. 

## 3. Results

The preparation of pectin–PLA hybrid aerogels required the optimization of several conditions. The choice of solvents and processing conditions was crucial to obtain a gel. Various solvents for PLA, such as chloroform, ethanol, and N-methyl-2-pyrrolidone, did not give stable gels after contact with an aqueous pectin solution. Ethyl lactate (EL) was chosen for further hybrid preparation, and the temperature had to be increased up to 120 °C to dissolve the PLA effectively. EL was already proven to be an effective solubilizing agent for PLA, resulting in thermal gel formation [28]. The aging of the prepared wet gels in ethanol was evaluated for three days, and it was shown that aging does not benefit the final material characteristics in terms of surface area. Therefore, all the prepared wet gels were dried supercritically the next day without additional aging in ethanol. The next step was adjusting the ratio between pectin and PLA, resulting in a series of P:PLA hybrid aerogels, designated as P1:PLA1, P1:PLA2, and P2:PLA1, where 1 and 2 are the weight ratio of the pectin and PLA solutions (Table 1). All the obtained hybrid aerogels appeared as white monoliths or thick films after supercritical drying, with densities ranging from 0.13–0.36 g·cm^−3^ (Table 2). As expected, the density increased with increasing PLA content.

**Figure 1:**
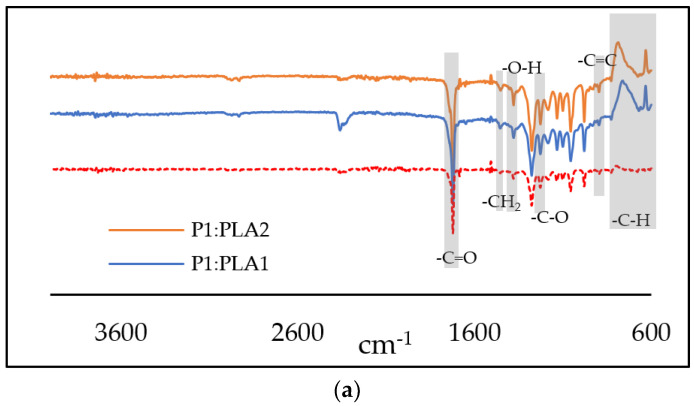

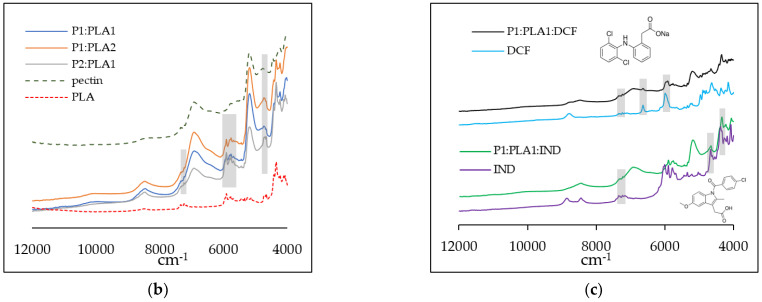
(**a**) FT-IR spectra of P1:PLA1, P1:PLA2, and PLA samples. (**b**) Comparison of FT–NIR spectra of the prepared hybrid samples and references, and (**c**) Comparison of the FT–NIR spectra of hybrid samples loaded with model drugs IND and DCF with the native spectra of pure model drugs.

The molecular structure was characterized further with FT-IR and FT–NIR spectroscopy. The FT-IR spectra of the reference and hybrid materials are shown in Figure 1a, showing the prominent absorption peaks of PLA and pectin. The characteristic peak of PLA at 1722 cm^−1^ is associated with the -C = O stretching vibrations, and the -C-O vibration is shown at 1275 cm^−1^. The peaks at 1454 and 1379 cm^−1^ could be assigned to the –CH_2_ scissoring and –OH bending vibration peaks of pectin, respectively [29]. The -C-H bending vibration of pectin is shown at 615 cm^−1^ and -C = C at 830 cm^−1^. Figure 1b shows the significant peaks of PLA and pectin in the hybrids. Notable peaks of PLA between 7264 and 7352 cm^−1^ and between 5720 and 5928 cm^−1^ are present in all hybrid samples. The stretching vibration of C-H, N-H, and O-H at 6950 cm^−1^ is typical for polysaccharides such as pectin, and was present in all hybrid samples. The NIR spectra were then recorded to observe the incorporation of DCF and IND, since the FT-IR method did not show any significant peaks of the drugs present in the hybrids, most probably due to the low amount of the drug coupled with the method’s characteristics. Figure 1c shows the NIR spectra of pure IND and DCF drugs, respectively. These spectra were compared directly to the hybrid P1:PLA1:IND and P1:PLA1:DCF spectra. The characteristic peaks of IND at 7240 cm^−1^, 4680 cm^−1^, and 4360 cm^−1^ were present in the hybrid sample, confirming the presence of IND in the P1:PLA1:IND. Similarly, significant peaks of DCF at 7384 cm^−1^, 6608 cm^−1^, and 5992 cm^−1^ were present in the hybrid sample of P1:PLA1:DCF.

**Figure 2:**
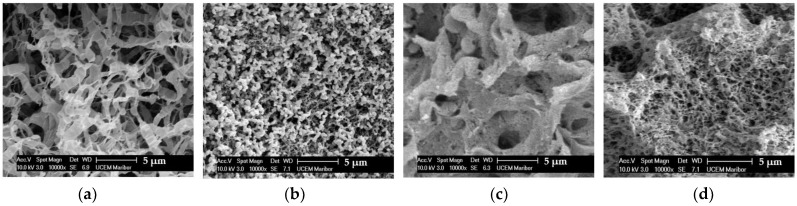
SEM images of (**a**) PLA, (**b**) Pectin, (**c**) P1:PLA2, (**d**) P1:PLA1.

The morphologies of the reference and hybrid materials are presented in Figure 2 and Figure S2. The PLA reference material is highly microporous (Figure 2a) and shows a fibrous-like structure. The referenced pectin aerogel shows a typical grape-like structure, already reported in the literature [30]. The P1:PLA2 hybrid sample shows a compact structure with larger voids, and it looks like pectin aerogel is entrapped inside the PLA skeleton. This structure is also visible in the P1:PLA1 sample, but with a higher pectin content, and, thus, a more mesoporous material structure. However, it is still visible that the pectin aerogel is entrapped between the PLA (Figure S2d, 1000 x magnification).

**Figure 3:**
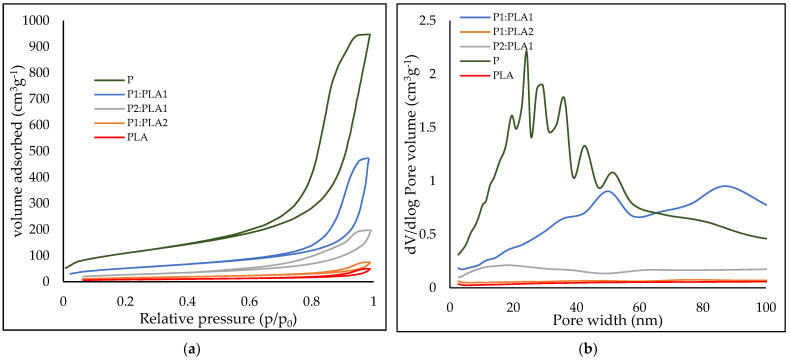
(**a**) Nitrogen physisorption curves and (**b**) Average mesopore size of the reference materials (P and PLA) and hybrids (P1:PLA1, P2:PLA1, and P1:PLA2).

The prepared reference and hybrid materials were characterized using nitrogen adsorption to determine their surface area and mean mesoporous size. Figure 3a compares the reference (P, PLA) and hybrid (P1:PLA1, P1:PLA2, and P2:PLA1) samples.

All the tested samples showed typical IUPAC IV adsorption/desorption hysteresis with H1 or H2 loops, which is typical for mesoporous materials. The highest adsorption capacity was observed in the pectin aerogel, which also had the highest mesopore volume (Figure 3b) and surface area of 386 m^2^·g^−1^. Among the hybrid materials, the highest adsorption capacity and mesopore volume were observed in the P1:PLA1 sample, and this sample provided the highest surface area of 166 m^2^·g^−1^. It would be expected that P2:PLA1 would have a higher surface area, adsorption capacity, and mesopore volume. It seems that the PLA, P1:PLA2, and P2:PLA1 samples exhibit accessible macroporosity. The low adsorption capacity of those samples and low mesoporosity result in a much lower surface area of those samples. The surface areas are 92, 37, and 29 m^2^·g^−1^ for P2:PLA1, P1:PLA2, and PLA samples, respectively. 

**Figure 4:**
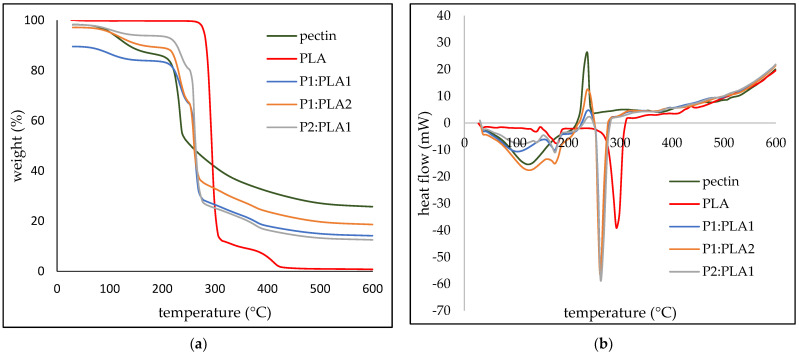
(**a**) TG and (**b**) DSC of the prepared hybrid and reference materials.

The degradation behavior of the hybrid materials and reference pectin and PLA aerogels is shown in Figure 4. The TG of pectin shows typical degradation after 220 °C. PLA aerogel starts to degrade after 290 °C, showing a significant melting peak. However, the hybrid materials show the usual combination of pectin and PLA degradation. The first degradation step at 220 °C for all three hybrid samples is associated with pectin degradation, followed by PLA degradation. In the case of hybrid materials, the degradation of PLA occurs at lower temperatures, with the melting peak at 260 °C, and not at 280 °C as shown for pure PLA. The reason for this phenomenon could be physical bonding of pectin and PLA. The hybrid materials were prepared by the combination of a PLA solution in EL with a pectin aqueous solution; thus, the gel formation was probably governed not only by the cooling step, but also by the antisolvent addition.

The TG and DSC of indomethacin and diclofenac sodium are shown in Figure S3, where it is shown that the melting of the indomethacin occurs at 160 °C, and the degradation starts at around 140 °C. The DCF shows a characteristic exothermic peak at around 288 °C, followed by an endothermic peak at around 310 °C. The degradation of DCS starts at around 290 °C, with 45% mass loss at 600 °C. 

The amount of pectin in the hybrid samples was determined through TG curves. The determined values of weight percent of pectin in the hybrid samples were 60.8%, 73.8%, and 49.1% for P1:PLA1, P2:PLA1, and P1:PLA2, respectively (Table 2).

**Figure 5:**
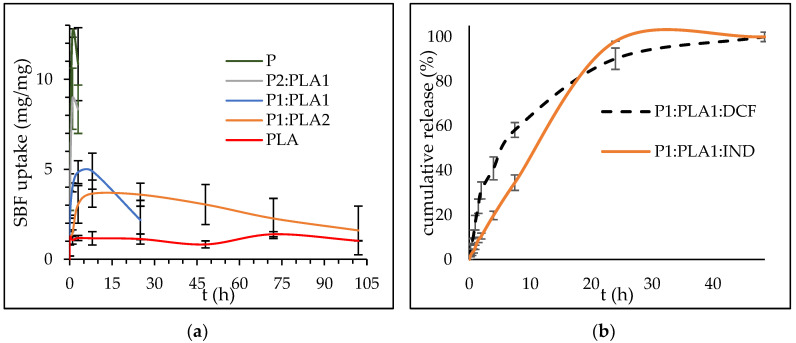
(**a**) SBF uptake of the reference and hybrid samples, (**b**) DCF and IND dissolution from the P1:PLA1:DCF and P1:PLA1:IND samples. Each sample was measured in duplicate, and the standard deviation is expressed as error bars.

After contact with a moist environment, hydrophilic aerogels may undergo degradation. Therefore, it is necessary to understand the degradation rate of the material that is intended for wound treatment to identify the time window for its actual application. In this case, the degradation of the aerogels was studied in simulated body fluid (SBF). As seen in Figure 5a, the degradation of pectin aerogel was the fastest among all the materials. This material also has a higher SBF uptake, up to 13 times its weight. Among hybrid materials, the quickest degradation occurred in the P2:PLA1 sample, the one with higher pectin content, which was expected. This material could hold up to ten times its weight in fluid. However, the degradation occurred after only 2 h, making this material unsuitable for wound application. The P1:PLA1 sample started to degrade after 24 h, and its SBF uptake was five times its weight. The lowest SBF uptake was observed in the PLA and P1:PLA2 samples, which were also the most stable ones. Those two samples were stable for more than one week. Due to its higher SBF uptake, the P1:PLA1 sample was chosen for drug incorporation. Two NSAID drugs, IND and DCF, were tested, and the drug dissolution results are shown in Figure 5b. It is seen that both drugs released from the material almost entirely after 24 h. The dissolution of DCF was faster in the initial step, whereas the IND release was slower and showed linear behavior. According to the NIR, there are no additional peaks generated from the P1:PLA1:IND and P1:PLA1:DCF samples; therefore, it is expected that there is no chemical bonding between the matrix and the drug. The initial fast release of DCF possibly occurs due to the higher hydrophilicity of this drug compared to IND, which is hydrophobic and thus its release is slower. However, the release of the IND is fast compared to the pure hydrophobic drug, indicating that the matrix (P1:PLA1) may influence the drug characteristics. The mesoporous structure of the hybrid materials may be beneficial for increasing the release rate of low-water-soluble drugs.

**Figure 6:**
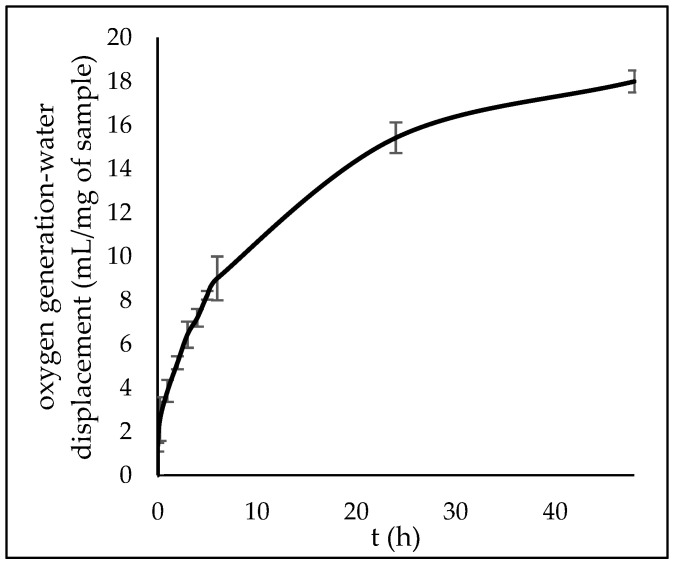
Oxygen generation from P1:PLA1:O_2_, measured for 2 days.

Oxygen generation from hybrid materials was tested (Figure 6) additionally, as it presents a novel and promising method for wound treatment. The aqueous environment of a wound initiates the decomposition of SPO and CPO to release oxygen [23]. Oxygen release studies over 2 days showed the initial burst generation of oxygen and exponential behavior up to 48 h.

## 4. Discussion

Aerogels show advanced and auspicious characteristics in terms of wound dressings. They can be synthesized from abundant polymers, such as polysaccharides or biopolymers, which are known for their low price. Additionally, supercritical technology allows processing under mild conditions, and produces materials that have properties of wet gels, which is of great importance, especially in biomedical applications. Due to their preserved porous structure, high porosity, and large surface area, aerogels can incorporate drugs and deliver them at the desired rate. It is a unique characteristic of aerogels that they can slow down the release of highly water-soluble drugs and improve the solubility and release of low-water-soluble drugs. This research was, thus, focused on the preparation of pectin–PLA hybrid aerogels using a straightforward method. 

Overall, this study describes the first-time preparation of pectin–PLA hybrid aerogels, their characterization, and their application in wound treatments. The P2:PLA1 sample had an expected higher swelling and faster degradation rate due to the higher pectin content. As mentioned in the introduction, PLA was chosen to stabilize the material, and, in combination with pectin, offered more increased swelling and simulated body fluid (SBF) uptake than pure PLA material. Even though the P1:PLA2 sample had low SBF uptake, its stability was prolonged for over two weeks. The P1:PLA1 sample with the same weight PLA and pectin solution started to degrade after 24h in contact with simulated body fluid. This material had a high surface area, and incorporated two model drugs, IND and DCF. Drug release is often controlled by the hydration of the polymeric matrix and its swelling behavior, then the diffusion through the matrix, and, finally, the erosion of the matrix. Since the wound exhibits a different amount of exudate, it is expected that the release can be achieved by the swelling and erosion of the material. The dissolution rate is then not controlled only by the matrix, but also by the drug’s properties. Therefore, incorporated into the same material, IND and DCF showed different dissolution profiles. The DCF, with higher water solubility, showed faster initial release; the low-solubility IND showed a slower, controlled release over 24h. Additionally, SPO and CPO, two common oxygen-generating materials that can improve wound healing and tissue regeneration [31,32,33], were incorporated into the P1:PLA1 sample to provide oxygen generation over a 2-day period. Oxygen was generated for 48 h while the sample was degrading, showing promising results in wound-healing applications.

## 5. Conclusions

This research reports a unique, straightforward method to produce pectin–PLA hybrid materials with a cotton-like structure. High water uptake and swelling ability makes such materials interesting in wound-dressing applications. The use of PLA significantly affects the stability of the hybrid materials in SBF. The prepared hybrid materials can incorporate various pharmaceuticals, independent of their water solubility. The release of two model drugs is governed by the matrix structure but also by their water solubility. Incorporation of the oxygen-generating agents showed prolonged release of oxygen from the pectin–PLA hybrids, which could positively affect wound healing. Therefore, the results of this study show very promising steps in the production of active wound-healing materials.

## Figures and Tables

**Table 1:** Sample composition.

Sample ^1^	Pectin (g)	PLA (g)	H_2_O (g)	EL (g)	IND (mg)	DCF (mg)	SPO (g)	CPO (g)
P1:PLA1	1.0	1.0	25	25	-	-	-	-
P2:PLA1	1.0	0.5	25	25	-	-	-	-
P1:PLA2	0.5	1.0	25	25	-	-	-	-
P	1.0	-	25	25	-	-	-	-
PLA	-	1.0	25	25	-	-	-	-
P1:PLA1:IND	1.0	1.0	25	25	500	-	-	-
P1:PLA1:DCF	1.0	1.0	25	25	-	500	-	-
P1:PLA1:O_2_	1.0	1.0	25	25	-	-	1.0	1.0

^1^ P: pectin; PLA: polylactic acid; EL: ethyl lactate; IND: indomethacin; DCF: diclofenac sodium; SPO: sodium percarbonate; CPO: calcium peroxide.

**Table 2:** Sample characterization.

Sample ^1^	S_BET_ (m^2^·g^−1^)	ρ [g·cm^−3^]	Pectin Content (%)	Degradation Start (h)	Drug Content (%)	Entrapment Efficiency (%)
P1:PLA1	166 ± 23	0.32 ± 0.02	60.8	25	-	-
P2:PLA1	92 ± 3	0.13 ± 0.02	73.8	3	-	-
P1:PLA2	37 ± 2	0.36 ± 0.01	49.1	102	-	-
P	386 ± 21	0.09 ± 0.002	100	3	-	-
PLA	29 ± 3	0.07 ± 0.01	0	102	-	-
P1:PLA1:IND	-	-	60.8	-	2.2 ± 0.6	63.4 ± 0.1
P1:PLA1:DCF	-	-	60.8	-	3.3 ± 0.4	62.6 ± 0.1

^1^ P: pectin; PLA: polylactic acid; EL: ethyl lactate; IND: indomethacin; DCF: diclofenac sodium.

## Data Availability

Not applicable.

## Supplementary Materials

The following supporting information can be downloaded at: https://www.mdpi.com/article/10.3390/polym15020407/s1, Figure S1: Modified supercritical extraction unit.; Figure S2: SEM picture of a) PLA, b) pectin, c) P1:PLA2, d) P1:PLA1; Figure S3: (a) TG and (b) DSC of indomethacin and diclofenac sodium.
