# Supplementary material for: Hybrid Polylactic-Acid–Pectin Aerogels: Synthesis, Structural Properties, and Drug Release
Source: Polymers (Basel). 2023 Jan 12;15(2):407. doi: 10.3390/polym15020407 (PMC9862002; doi:10.3390/polym15020407)
Supplement: Supplementary file 1 [file polymers-15-00407-s001.zip › polymers-2148919-supplementary.pdf]

polymers

MDPI

Supplementary Information

Hybrid polylactic acid-pectin aerogels: synthesis, structural properties, and drug release

Gabrijela Horvat [1], Klara Žvab [1], Željko Knez[1] and Zoran Novak [1,*]

[1] University of Maribor, Faculty of Chemistry and Chemical Engineering, Smetanova 17, SI-2000 Maribor, Slovenia
* Correspondence: Zoran.novak@um.si;

1. Shematic presentation of supercritical extraction unit

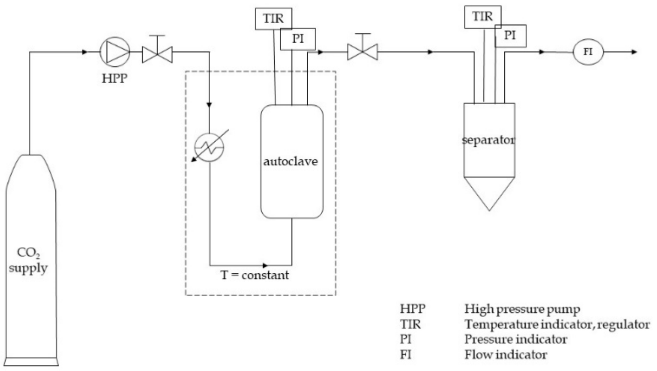

Figure S1. Modified supercritical extraction unit.

2. SEM

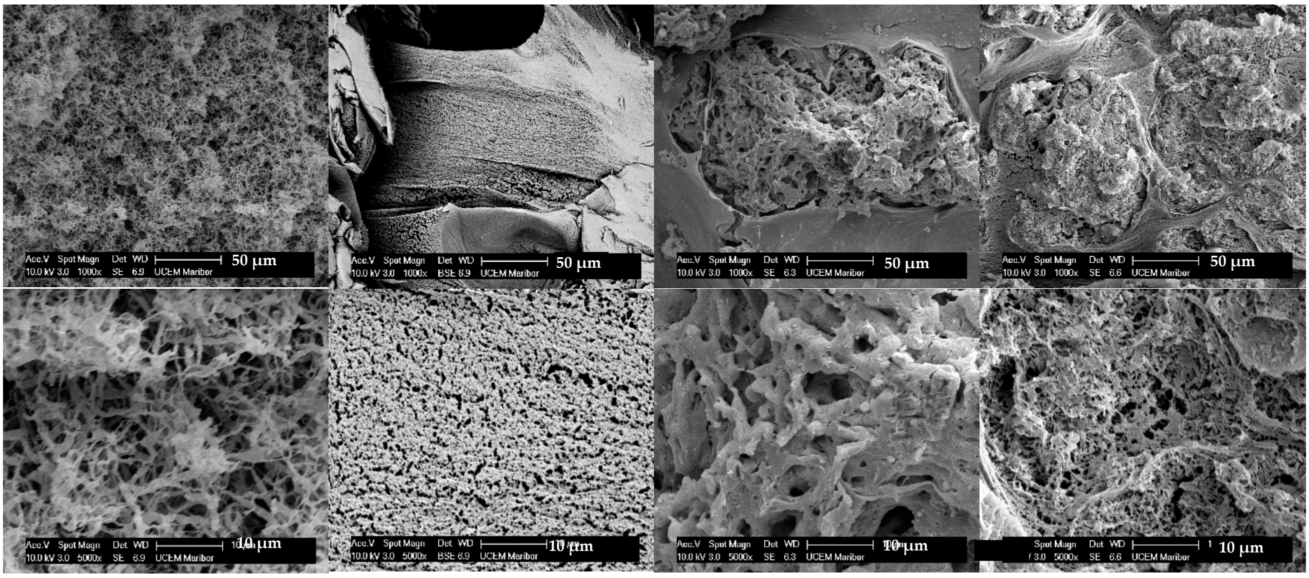

Figure S2. SEM picture of a) PLA, b) pectin, c) P1:PLA2, d) P1:PLA1

3. TG and DSC analysis of the drugs

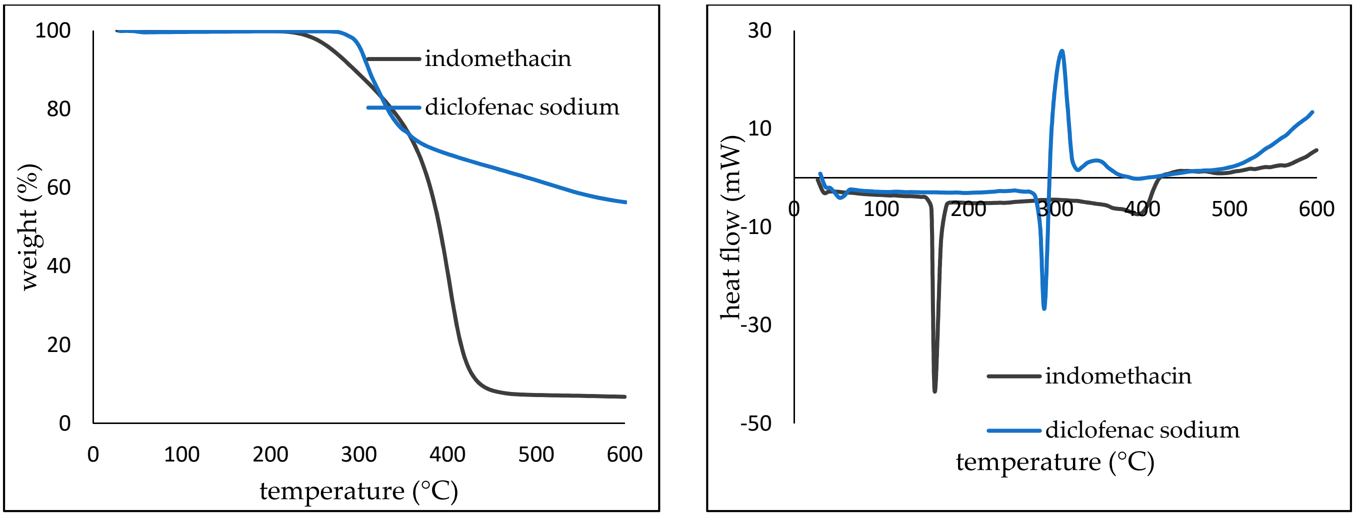

(a) (b)

Figure S3. a) TG and b) DSC of indomethacin and diclofenac sodium.
